# Outcome measures in randomized controlled trials of patients with severe traumatic brain injury: a systematic review

**DOI:** 10.1186/cc13668

**Published:** 2014-03-17

**Authors:** G Lalonde', M Shemilt, F Lauzier, P Desjardins, A Boutin, L Moore, D Fergusson, R Zarychanski, A Turgeon

**Affiliations:** 1 Laval University, Quebec, Canada; 2 OHRI, Ottawa, Canada; 3 University of Manitoba, Winnipeg, Canada

## Introduction

Traumatic brain injury (TBI) is a major cause of death and long-term disability worldwide, and understanding its effect on health is critical. Although mortality has been a gold standard for years, functional scales and quality of life have been used as main outcome measures over the last decades due to their usefulness and aid in decision-making for medical teams, patients, and families. Despite this preference, no consensus exists for the most optimal outcome measure. We systematically reviewed outcome measures used in randomized controlled trials (RCTs) in patients with severe TBI admitted to an acute care hospital.

## Methods

We searched MEDLINE, EMBASE, Cochrane Central, BIOSIS and references of eligible trials. RCTs published over the last 7 years (2006 to October 2013) in 18 selected journals (based on impact factor) were considered for inclusion. RCTs performed in adults with severe TBI (GCS ≤8) were eligible. The primary endpoint was the outcome measures used in RCTs. The secondary outcomes were the timing of assessment and the methodological quality of trials using the Cochrane risk of bias assessment tool. Two independent reviewers selected trials and collected data using a standardized form.

## Results

From 5,602 citations retrieved after removal of duplicates, 36 RCTs met eligibility criteria. The outcome measures most frequently used were neurophysiologic indices (*n *= 18, 50%), the Glasgow Outcome Scale (*n *= 17, 47%), mainly at 6 months, nonspecific complications (*n *= 15, 42%), mortality (*n *= 12, 33%), and biomarkers (*n *= 10, 28%). Nine trials reported only physiologic indices and did not present any clinical or functional outcome measures. The methodological quality of included RCTs was heterogeneous. We observed a low risk of bias for sequence generation (*n *= 29, 80%), allocation concealment (*n *= 20, 55%), complete data reporting *(n *= 30, 83%), selective reporting (*n *= 36, 100%), and sample size (*n *= 21, 58%) but a high risk of bias for blinding *(n *= 20, 55%).

## Conclusion

Outcome measures used to evaluate the effect of intervention in RCTs performed in patients with severe TBI in acute care are heterogeneous. A significant proportion of trials did not consider evaluating the functional status or other clinically meaningful outcomes, and very few trials assessed outcomes beyond 6 months. Thus, a significant proportion of RCTs in patients with severe TBI are based on outcome measures not clinically useful to guide or change practice in this population.

